# Maternal Serum Lipid Levels in the Third Trimester and Associated Factors in Vietnam

**DOI:** 10.1155/ogi/8389320

**Published:** 2026-03-04

**Authors:** Nguyen Viet Ha, Pham Manh Hung, Pham Ba Nha, Nguyen Tien Hoang

**Affiliations:** ^1^ Department of Obstetrics and Gynecology, Hanoi Medical University, Hanoi, Vietnam, hmu.edu.vn; ^2^ Department of Cardiology, Hanoi Medical University, Hanoi, Vietnam, hmu.edu.vn; ^3^ Women’s Health Center, Vinmec Times City International Hospital, Hanoi, Vietnam

**Keywords:** cholesterol, HDL, LDL, pregnancy, third trimester, triglycerides

## Abstract

**Introduction:**

There is a lack of studies on lipid levels in Vietnamese pregnant women. Our study aimed to describe serum lipid levels of healthy Vietnamese women in the third trimester and identify factors influencing these levels.

**Method:**

A cross‐sectional study on 1022 healthy females with singleton pregnancy intended to deliver at Bach Mai Hospital recruited from April 2023 to June 2024. Measure fasting serum total cholesterol (TC), low‐density lipoprotein cholesterol (LDL‐C), high‐density lipoprotein cholesterol (HDL‐C), and triglycerides at 28–40 weeks of gestation by enzymatic colorimetric assays. Assess factors associated with maternal lipid levels by multivariable linear regression.

**Results:**

Median (95% reference range) of TC, LDL‐C, HDL‐C, and triglycerides were 6.48 (4.39–8.88), 3.33 (1.59–5.52), 1.81 (1.25–2.53), 2.90 (1.65–6.06) mmol/L, respectively. Complicated pregnancy (*n* = 377) had higher triglycerides and lower cholesterol levels than the uncomplicated pregnancy (*n* = 645). Factors associated with TC were prepregnancy BMI and gestational age. For LDL‐C: prepregnancy BMI, gestational age, and gestational diabetes mellitus (GDM). For HDL‐C: prepregnancy BMI and GDM. For TG: prepregnancy BMI, gestational age, GDM, a history of hypertensive disorder in pregnancy, and a history of macrosomia.

**Conclusion:**

We presented 95% reference range for the third trimester serum lipid levels in healthy Vietnamese women. Obstetric complications were associated with decreased LDL‐C and HDL‐C and increased TG, along with gestational age and prepregnancy BMI.

## 1. Introduction

Serum lipid levels have been reported to change dramatically during pregnancy, especially in the third trimester. Lipid concentrations begin to rise at the 12th week of gestation and by the end of pregnancy, the increase of total cholesterol (TC) level ranges from 30% to 50%, while the number for triglyceride (TG) level is 200% to 400% [1]. The mechanism of these alterations is increasing insulin resistance in late pregnancy, which stems from the increased production of human placental lactogen, cortisol, estrogen, adipokines, and tumor necrosis factor‐alpha by the fetoplacental unit [2]. Insulin resistance shifts maternal metabolism to fatty acid oxidation to preserve glucose for fetal supply. On the other hand, insulin resistance downregulates TG uptake and facilitates lipolysis which produces more free fatty acids (FFAs) and glycerol than the demand of total body fat oxidation [3]. The excess FFA and glycerol are subsequently processed by liver into ketone bodies and TG which are packaged into VLDL particles [4]. Maternal FFAs serve as a supplemental source of polyunsaturated fatty acids for fetal brain development in the third trimester [2].

Although maternal hyperlipidemia is considered a physiological process, increasing evidence indicates significant associations between this condition and adverse pregnancy outcomes including gestational diabetes mellitus (GDM), hypertensive disorders of pregnancy (HDP), large‐for‐gestational age (LGA), small‐for‐gestational age (SGA) newborns and pancreatitis [5]. Elevated blood lipid levels in pregnancy also increase the risk of long‐term hypertension [6] and offspring adiposity [7].

Several studies have been conducted to determine the normal range of cholesterol and TG in the last trimester by various methods, mostly in Chinese and European population [8–14]. However, most of them emphasized the fluctuations of serum lipid level as pregnancy advances and there is a need for further research to explore the role of other factors. Moreover, there has been no study on serum lipid concentrations among Vietnamese pregnant women in the third trimester. Therefore, our study aimed to describe the serum lipid levels of healthy Vietnamese women in the third trimester and identify factors associated with these concentrations.

## 2. Materials and Methods

### 2.1. Study Design

This cross‐sectional study was conducted in Bach Mai Hospital, Hanoi, Vietnam. Bach Mai Hospital is a leading tertiary‐level general hospital in northern Vietnam, and its Department of Obstetrics and Gynecology is responsible for providing comprehensive care for women during pregnancy, childbirth, and the postpartum period. The department managed an average of 4300 deliveries annually.

### 2.2. Participants

Participants included healthy pregnant women aged 18–45 years who attended prenatal care and intended to deliver at the Department of Obstetrics and Gynecology of Bach Mai Hospital. These women were recruited between April 2023 and June 2024 using convenience sampling. Inclusion criteria were as follows: (1) gestational age 28–40 weeks; (2) singleton pregnancy; and (3) natural conception. The participants were excluded if they had preexisting chronic diseases, including hypertension, diabetes mellitus, thyroid, kidney, or cardiovascular diseases. Women with a history of tobacco or alcohol exposure during pregnancy, inherited metabolic diseases, or fetal anomalies were also excluded. The required sample size was calculated based on the estimation of a single mean. The standard deviations (SDs) of serum lipid levels at 28, 36, and term gestational weeks were derived from the study by Jimenez et al. [15]. For each gestational age group (28–32, 33–36, and 37–40 weeks), sample size was calculated separately using the corresponding SDs for TC, low‐density lipoprotein cholesterol (LDL‐C), high‐density lipoprotein cholesterol (HDL‐C), and TG , with an allowable error of 12% of the SD and a 95% confidence level. The largest estimated sample size among the four lipid parameters was selected for each gestational group, which resulted in 267 participants. Considering a 10% allowance for potential data loss or exclusions, the final target sample size was set at 294 participants per gestational group. Accordingly, the total estimated sample size for the study was 882 participants. However, a total of 1022 pregnant women were enrolled, including 329, 373, and 320 pregnant women in the 28–32, 33–36, and 37–40 weeks gestational age groups, respectively.

### 2.3. Data Collection

At study entry, all recruited participants were requested to complete a questionnaire regarding maternal age, height, prepregnancy weight, residence, a history of obstetric complications and GDM. Blood samples were collected to measure concentrations of TC, LDL‐C, HDL‐C, and TG. At delivery, we obtained data regarding gestational weight gain (GWG), HDP, delivery mode, gestational age, newborn sex, birthweight, and 5‐min postpartum Apgar score.

### 2.4. Biochemical Analyses

Venous blood samples were collected after overnight fasting and analyzed within 2 h of collection at ambient temperature (20°C–25°C), without freezing or refrigeration. Serum TC, TG, HDL‐C, and LDL‐C were determined using enzymatic colorimetric methods on a Beckman Coulter AU5800 autoanalyzer (Beckman Coulter, Brea, CA, USA) in the ISO 15189:2012‐accredited Biochemistry Laboratory, Bach Mai Hospital.

TC, TG, HDL‐C, and LDL‐C were measured with Beckman Coulter reagents AU Cholesterol (OSR6116), AU Triglyceride (OSR61118), AU HDL‐Cholesterol (OSR6287), and AU LDL‐Cholesterol (OSR6183), respectively. Calibration was performed using the System Calibrator (Cat. No. 66300) traceable to National Institute of Standards and Technology (NIST) Standard Reference Material (SRM) 909b and Centers for Disease Control and Prevention (CDC) reference methods. Two levels of control sera (ODC0003 and ODC0004) were run daily, and all assays met the manufacturer’s specifications for linearity and precision (coefficient of variation < 2%).

All samples were visually inspected for hemolysis, lipemia, and icterus prior to testing according to standard laboratory procedures. No specimens exhibited significant interference, and all were included in the analysis.

### 2.5. Definitions

Body mass index (BMI) was calculated by dividing weight in kilograms by the square of height in meters. Maternal prepregnancy BMI was categorized into underweight (< 18.5 kg/m^2^), normal weight (18.5–24.9 kg/m^2^), overweight (25.0–29.9 kg/m^2^), and obese (≥ 30.0 kg/m^2^) groups according to the World Health Organization BMI classification [16].

GWG was stratified into appropriate, excessive, and inadequate groups based on the American Institute of Medicine [17], in which appropriate GWG was defined as 12.5–18.0 kg in underweight women, 11.5–16.0 kg in normal weight women, 7.0–11.5 kg in overweight women, and 5.0–9.0 kg in obese women. GWG greater than the thresholds was defined as excessive, while values lower than the thresholds were defined as inadequate GWG.

HDP included gestational hypertension and preeclampsia, which were defined as blood pressure ≥ 140 mm·Hg systolic or 90 mm·Hg diastolic on at least two occasions 4–6 h apart on or after 20 weeks gestational age, with or without proteinuria (300 mg protein or more in a 24‐h urine sample or positive on a urine dipstick) in previously normotensive women [18].

We used criteria from the International Association of Diabetes and Pregnancy Study Groups to determine GDM with a one‐step oral glucose tolerance test performed at 24–28 weeks of gestation. GDM was diagnosed if any of the following venous plasma glucose thresholds were reached or surpassed: fasting 5.1 mmol/L, 1‐h postload 10.0 mmol/L, and 2‐h postload 8.5 mmol/L [19].

Newborn weight was classified as appropriate for gestational age, SGA, and LGA based on standards from the International Fetal and Newborn Growth Consortium for the 21st Century (INTERGROWTH‐21st). Neonates were defined as SGA if their birth weights fell under the 10th percentile and LGA if their birth weights exceeded the 90th percentile for gestational age and sex. AGA was defined as birth weights between the 10th and 89th percentile for gestational age and sex. Newborns were considered macrosomic if their birth weights were 4000 g or more. Complicated pregnancy was defined as having at least one complication including HDP, GDM, preterm birth, SGA, LGA, and macrosomia.

### 2.6. Statistical Analysis

All statistical analyses were performed using IBM SPSS Statistics, Version 26.0 (IBM Corp., Armonk, NY, USA). Continuous variables were expressed as mean ± SD or median (interquartile range [IQR]) as appropriate, and categorical variables as frequencies and percentages. Group comparisons were conducted using Student’s *t*‐test, the Mann–Whitney *U* test, Kruskal‐Wallis test, or the *χ* ^2^ test, depending on data distribution.

To identify independent factors associated with maternal serum lipid levels, four separate multivariable linear regression models were constructed, with TC, LDL‐C, HDL‐C, and TG as dependent variables. Independent variables included those with biological plausibility and/or statistical significance in univariate analyses: maternal age, place of residence (urban/rural), prepregnancy BMI, a history of macrosomic neonate, a history of hypertensive disorders in previous pregnancies, gestational age at examination, and GDM. Variables considered as pregnancy outcomes occurring after lipid measurements—such as mode of delivery, neonatal LGA, GWG and HDP—were excluded to avoid overadjustment or collider bias.

Before model fitting, all variables were checked for missing data, outliers, and data‐entry errors. Potential outliers were examined by standardized residuals, leverage values, Mahalanobis distance, and Cook’s distance. No influential outliers were detected, and extreme values related to data‐entry errors were corrected. As TG concentrations a showed right‐skewed distribution, we assessed the impact of logarithmic transformation; however, the residual diagnostics demonstrated satisfactory normality and homoscedasticity without transformation, so raw values were used for interpretability.

Model assumptions of linear regression—including linearity, independence of errors, normality, homoscedasticity, and multicollinearity—were assessed for all four models. Linearity and homoscedasticity were verified by inspection of residual‐versus‐predicted plots, normality by histogram and P–P plot of standardized residuals, and independence of residuals by the Durbin–Watson statistic. Multicollinearity was evaluated by variance inflation factor (VIF). All four models satisfied these assumptions, with no evidence of autocorrelation or heteroscedasticity (Durbin–Watson ≈ 2.0; all VIF < 2.0). Results were reported as unstandardized regression coefficients (B) with 95% confidence intervals (95% CIs) and *p* values. Statistical significance was defined as two‐tailed *p* < 0.05.

A secondary analysis of this cohort examining maternal lipid levels as predictors of GDM has been previously posted as a preprint on medRxiv [20]. The present study addresses a different research objective, focusing on establishing reference ranges and identifying factors associated with maternal lipid levels.

### 2.7. Ethical Consideration

The study protocol was approved by Hanoi Medical University Institutional Ethical Review Board (IRB00002121) (Certificate of Approval No. 833/GCN‐HĐĐĐNCYSH‐ĐHYHN). All participants provided written informed consent after receiving a clear and comprehensive explanation of the study’s objectives, procedures, potential risks, benefits, and their right to withdraw at any time without penalty.

## 3. Results

Maternal and neonatal characteristics are presented in Table 1. The mean (±SD) maternal age was 29.85 ± 4.90 years, with approximately 70% of participants aged 25–34 years. About 8% of participants were overweight or obese, while 14% of them were underweight before pregnancy. The percentage of women with a history of macrosomic neonates and HDP in previous pregnancies was 2.4% and 1.7%, respectively. The distribution of gestational age at examination was relatively even across 3 groups, with mean ± SD was 34.36 ± 2.77 weeks. Excessive GWG accounted for 18.4% of participants, while inadequate GWG was observed at 39.6%. The prevalence of HDP and GDM were 3.1% and 26.5%, respectively. Regarding neonates, the mean gestational age at delivery was 38.79 weeks, with a preterm birth rate of 4.6%. Low birth weight and macrosomia accounted for 2.8% and 2.3%, respectively. Stratified for gestational age at delivery and newborn gender, 4.0% of them were SGA, 88.8% were AGA, and 7.2% were LGA.

**TABLE 1:** Maternal and neonatal characteristics.

Characteristics	Mean ± SD or *N* (%)
Maternal characteristics	
Maternal age at recruitment (years)	29.85 ± 4.90
18–24	142 (13.9)
25–34	709 (69.4)
35 and older	171 (16.7)
Residence	
Rural	520 (50.9)
Urban	502 (49.1)
Prepregnancy BMI (kg/m^2^)	21.15 ± 2.73
Underweight (< 18.5 kg/m^2^)	143 (14.0)
Normal weight (18.5–24.9 kg/m^2^)	802 (78.5)
Overweight and obese (≥ 25 kg/m^2^)	77 (7.5)
History of macrosomic neonates	25 (2.4)
History of HDP	17 (1.7)
Gestational age at examination (weeks)	34.36 ± 2.77
28–32	329 (32.2)
33–36	373 (36.5)
37–40	320 (31.3)
Gestational weight gain	
Inadequate	405 (39.6)
Appropriate	429 (42.0)
Excessive	188 (18.4)
Hypertension disorders in pregnancy	32 (3.1)
Gestational hypertension	20 (1.9)
Preeclampsia	12 (1.2)
Gestational diabetes mellitus	271 (26.5)
Delivery mode	
Vaginal	392 (38.4)
Cesarean section	630 (61.6)
Neonatal characteristics	
Gestational age at delivery (weeks)	38.79 ± 1.21
Preterm	47 (4.6)
Term	975 (95.4)
Newborn gender	
Male	567 (55.5)
Female	455 (44.5)
Birth weight (gr)	3202.15 ± 390.08
< 2500 gr	29 (2.8)
2500–3999 gr	969 (94.9)
Macrosomia	24 (2.3)
Weight for gestational age and gender	
SGA	41 (4.0)
LGA	74 (7.2)
AGA	907 (88.8)

*Note:* SGA/LGA/AGA, small/large/appropriate for gestational age.

Abbreviations: BMI, body mass index; HDP, hypertension disorders in pregnancy.

Table 2 summarizes the distribution of maternal serum lipid concentrations in the third trimester. The median (95% reference range) values were 6.48 (4.39–8.88) mmol/L for TC, 3.33 (1.59–5.52) mmol/L for LDL‐C, 1.81 (1.25–2.53) mmol/L for HDL‐C, and 2.90 (1.65–6.06) mmol/L for TG. Compared with women with uncomplicated pregnancies, those with adverse pregnancy outcomes had significantly lower concentrations of TC, LDL‐C, and HDL‐C, and higher TG levels.

**TABLE 2:** Serum lipid panel of Vietnamese pregnant women in the third trimester.

Serum level (mmol/L)	All participants (*N* = 1022)	Uncomplicated (*N* = 645)	Complicated (*N* = 377)	*p* value
Total cholesterol	6.48 (4.39–8.88)	6.55 (4.42–8.80)	6.37 (4.37–9.22)	**0.02** ^∗^
LDL‐cholesterol	3.33 (1.59–5.52)	3.42 (1.67–5.52)	3.20 (1.55–5.52)	**< 0.01** ^∗^
HDL‐cholesterol	1.81 (1.25–2.53)	1.83 (1.31–2.57)	1.78 (1.19–2.51)	**< 0.01** ^∗^
Triglycerides	2.90 (1.65–6.06)	2.77 (1.63–5.56)	3.15 (1.69–6.52)	**< 0.01** ^∗^

*Note:* Serum level presented as median (95% reference range). Bold values indicate statistically significant differences (*p* < 0.05).

^∗^Compare serum level between complicated and uncomplicated participants using the Mann–Whitney *U* test.

Prepregnancy factors associated with serum lipid levels are displayed in Table 3. Prepregnancy BMI was significantly associated with all four lipid parameters in the third trimester, showing a positive correlation with TG and inverse associations with TC, LDL‐C, and HDL‐C. Women with a history of macrosomic newborns or HDP exhibited significantly elevated levels of TG. Regarding LDL‐C, advanced maternal age, and a history of macrosomia were associated with lower level of this parameter; however, these differences were not statistically significant.

**TABLE 3:** Serum lipid level and prepregnancy factors.

Factors	Total cholesterol (mmol/L)	LDL‐cholesterol (mmol/L)	HDL‐cholesterol (mmol/L)	Triglycerides (mmol/L)
Maternal age (years)^∗∗^				
18–24	6.56 (1.53)	3.52 (1.16)	1.79 (0.41)	2.78 (1.14)
25–34	6.52 (1.50)	3.33 (1.29)	1.81 (0.41)	2.89 (1.25)
35 and older	6.37 (1.52)	3.28 (1.41)	1.83 (0.40)	3.00 (1.33)
*p* value	0.39	0.08	0.66	0.12
Residence^∗^				
Rural	6.50 (1.57)	3.37 (1.30)	1.82 (0.42)	2.93 (1.31)
Urban	6.43 (1.45)	3.31 (1.25)	1.81 (0.41)	2.88 (1.22)
*p* value	0.78	0.64	0.79	0.50
Prepregnancy BMI (kg/m^2^)^∗∗^				
< 18.5	6.63 (1.62)	3.65 (1.40)	1.85 (0.40)	2.65 (1.13)
18.5–24.9	6.49 (1.51)	3.30 (1.29)	1.81 (0.41)	2.91 (1.29)
25 or above	6.27 (1.61)	3.19 (1.40)	1.72 (0.43)	3.12 (1.22)
*p* value	**0.01**	**<** **0.01**	**0.02**	**<** **0.01**
History of macrosomic neonate^∗^				
Yes	6.15 (1.25)	3.08 (1.20)	1.78 (0.55)	3.46 (2.00)
No	6.49 (1.52)	3.36 (1.30)	1.81 (0.41)	2.88 (1.25)
*p* value	0.06	0.07	0.50	**0.01**
History of HDP^∗^				
Yes	6.53 (1.38)	3.21 (1.15)	1.73 (0.55)	3.89 (1.95)
No	6.47 (1.51)	3.33 (1.31)	1.82 (0.41)	2.88 (1.25)
*p* value	0.82	0.45	0.45	**0.01**

*Note:* Serum level presented as median (IQR). Bold values indicate statistically significant differences (*p* < 0.05).

^∗^Compare differences by the Mann–Whitney test.

^∗∗^Compare differences by the Kruskal–Wallis test.

Table 4 shows the pregnancy factors related to the serum lipid panel in the third trimester. Pregnant women complicated with GDM had significantly higher concentrations of TG and lower concentrations of all cholesterol levels. TC and LDL‐C as well as TG levels elevated as gestational age advanced, except for HDL‐C. TG levels were also significantly higher in pregnant women with HDP and C‐section. Women who delivered LGA newborns had elevated TG levels and decreased HDL‐C levels in their third trimester.

**TABLE 4:** Serum lipid level and pregnancy factors.

Factors	Total cholesterol (mmol/L)	LDL‐cholesterol (mmol/L)	HDL‐cholesterol (mmol/L)	Triglycerides (mmol/L)
Gestational age at examination^∗∗^				
28–32 weeks	6.34 (1.60)	3.30 (1.46)	1.83 (0.47)	2.77 (1.18)
33–36 weeks	6.35 (1.50)	3.30 (1.13)	1.81 (0.40)	2.87 (1.15)
37–40 weeks	6.68 (1.45)	3.44 (1.29)	1.82 (0.37)	3.06 (1.49)
*p* value	**< 0.01**	**0.02**	0.54	**< 0.01**
GDM^∗^				
Yes	6.32 (1.66)	3.07 (1.37)	1.74 (0.45)	3.26 (1.50)
No	6.55 (1.47)	3.43 (1.28)	1.83 (0.38)	2.82 (1.13)
*p* value	**< 0.01**	**< 0.01**	**< 0.01**	**< 0.01**
Gestational weight gain^∗∗^				
Inadequate	6.52 (1.61)	3.42 (1.40)	1.78 (0.40)	2.88 (1.24)
Appropriate	6.47 (1.42)	3.30 (1.24)	1.82 (0.42)	2.92 (1.32)
Excessive	6.35 (1.54)	3.22 (1.35)	1.86 (0.40)	2.87 (1.42)
*p* value	0.78	0.19	**0.03**	0.39
HDP^∗^				
Yes	6.50 (1.85)	3.09 (1.24)	1.80 (0.52)	3.55 (1.92)
No	6.48 (1.51)	3.35 (1.30)	1.82 (0.41)	2.87 (1.25)
*p* value	0.98	0.18	0.41	**< 0.01**
Delivery mode^∗^				
Vaginal	6.50 (1.43)	3.39 (1.39)	1.82 (0.41)	2.78 (1.15)
Cesarean section	6.46 (1.53)	3.33 (1.26)	1.81 (0.43)	2.95 (1.36)
*p* value	0.51	0.20	0.32	**0.01**
LGA neonate^∗^				
Yes	6.49 (1.72)	3.28 (1.21)	1.77 (0.47)	3.29 (1.84)
No	6.47 (1.50)	3.33 (1.31)	1.82 (0.41)	2.87 (1.25)
*p* value	0.87	0.84	**0.04**	**< 0.01**

*Note:* Serum level presented median (IQR). Bold values indicate statistically significant differences (*p* < 0.05).

^∗^Compare differences by the Mann–Whitney test.

^∗∗^Compare differences by the Kruskal–Wallis test.

In multivariable analyses (Table 5), prepregnancy BMI was inversely associated with TC, LDL‐C, and HDL‐C but positively correlated with TG. Gestational age at examination showed a positive association with TC, LDL‐C, and TG, except for HDL‐C. GDM was independent factor of higher TG levels, whereas it was associated with lower LDL‐C and HDL‐C. A history of macrosomic neonate or HDP was additionally linked to elevated TG concentrations.

**TABLE 5:** Multivariable linear regression model for serum lipid in the third trimester.

Factors	Total cholesterol		LDL‐cholesterol		HDL‐cholesterol		Triglycerides	
	Coefficient (95% CI)	*p* value	Coefficient (95% CI)	*p* value	Coefficient (95% CI)	*p* value	Coefficient (95% CI)	*p* value
Maternal age (years)	0.00 ((‐0.02)–0.01)	0.80	−0.01 ((‐0.02)–0.01)	0.32	0.00 ((‐0.01)–0.01)	0.10	0.00 ((‐0.01)–0.02)	0.70
Residence (urban vs. rural)	−0.01 ((‐0.15)–0.14)	0.92	0.00 ((‐0.12)–0.13)	0.93	−0.01 ((‐0.05)–0.03)	0.62	−0.02 ((‐0.16)–0.11)	0.74
Prepregnancy BMI (kg/m^2^)	**−0.06 ((-0.09)–(-0.03))**	**< 0.01**	**−0.05 ((-0.08)–(-0.03))**	**< 0.01**	**−0.01 ((-0.02)–(-0.01))**	**< 0.01**	**0.03 (0.01–0.06)**	**0.01**
History of macrosomic neonate (yes vs. no)	−0.35 ((‐0.81)–0.11)	0.14	−0.26 ((‐0.65)–0.14)	0.20	−0.05 ((‐0.18)–0.08)	0.45	**0.44 (0.01–0.89)**	**0.04**
History of HDP (yes vs. no)	0.08 ((‐0.48)–0.64)	0.77	−0.01 ((‐0.49)–0.46)	0.96	−0.01 ((‐0.16)–0.14)	0.89	**0.69 (0.15–1.23)**	**0.01**
Gestational age at examination (weeks)	**0.04 (0.02–0.07)**	**0.01**	**0.02 (0.01–0.05)**	**0.03**	0.00 ((‐0.01)–0.01)	0.34	**0.08 (0.05–0.10)**	**< 0.01**
GDM (yes vs. no)	−0.11 ((‐0.28)–0.05)	0.18	**−0.18 ((-0.33)–(-0.04))**	**0.01**	**−0.07 ((-0.11)–(-0.02))**	**0.01**	**0.43 (0.28–0.59)**	**< 0.01**

*Note:* Bold values indicate statistically significant differences (*p* < 0.05).

## 4. Discussion

In our population‐based prospective study, we presented the 95% reference range of serum TC, LDL‐C, HDL‐C, and TG levels of healthy Vietnamese women in their third trimester of pregnancy. Women with pregnancy complications exhibited significantly higher TG levels and lower TC, LDL‐C, and HDL‐C levels, compared to those without complications. The study also revealed factors related to the lipid profile. TC concentration was associated with prepregnancy BMI and gestational age at sampling. LDL‐C level shared the same factors, plus GDM. For HDL‐C, prepregnancy BMI and GDM were related to decreased levels. Factors associated with increased TG levels were prepregnancy BMI, gestational age, GDM, a history of HDP, and a history of macrosomic neonates. All these associations were confirmed after adjustment for potential confounders in multivariable linear regression models.

Serum lipid concentrations during the third trimester were markedly elevated compared with the normal ranges reported in nonpregnant women, reflecting the physiological hyperlipidemia of late pregnancy. Increasing insulin resistance in late gestation, mediated by placental hormones [21] and adipokines [22], shifts maternal energy utilization toward fatty acid oxidation and promotes hepatic TG synthesis. Following a slight decrease in the second gestational month, TC, LDL‐C, and TG levels progressively increased and reached a peak at the delivery month. The rate of elevation in TC and LDL‐C during the third trimester was less pronounced when compared to the rate of increase in TG. HDL‐C concentration peaked at the 7th month and remained unchanged until delivery [23]. This physiological pattern explains the trends observed in our study, in which all lipid parameters except HDL‐C increased during the third trimester.

Beyond physiological changes, many authors reported the factors influencing maternal lipid levels and the magnitude of these changes during pregnancy, including prepregnancy BMI, diet, and ethnicity. Firstly, the association between prepregnancy BMI and maternal lipid levels in our study is consistent with other research on American and European women [24–28]. Overweight and obese women showed slower rates of TC and LDL‐C[25] despite higher TC, LDL‐C, and lower HDL‐C levels at early pregnancy [26, 28], which resulted in lower cholesterol concentration in the third trimester. The rate of TG increase during pregnancy was found to be comparable across prepregnancy BMI groups [25]; therefore, overweight and obese females had higher TG levels in the third trimester, compared with those of normal weight. During normal pregnancy, the gradual increase in TG‐rich lipoprotein enhances TG transfer to LDL in exchange for cholesteryl esters, mediated by cholesterol ester transfer protein. Sattar et al. reported that LDL remodeling depended on a specific TG threshold of each individual [29]. For this reason, these differences in lipid panels across prepregnancy BMI groups may be associated with metabolic dysregulation in overweight and obese gravidas. Secondly, cholesterol‐lowering diet from the second trimester was reported to decrease TC and LDL‐C in pregnant women [30]. Lastly, East Asian origin was also related to lower LDL‐C levels than Western European one in the third trimester [31].

Most studies on the reference range of serum lipid in the third trimester have been conducted among Chinese and European pregnant women without obstetric complications. Differences in maternal characteristics may partly explain the variability in lipid levels reported. Earlier gestational age of participants enrolled in studies of Dathan‐Stumpf (30.8 ± 4.4 weeks) [32] and Jin (29 ± 4 weeks) [11] than in our study could result in their lower range of TG level. Our TG result is consistent with studies including a wide range of gestational age in the third trimester [9, 12]. Besides, German pregnant women showed elevated TC and LDL‐C levels [32] compared to our findings and those reported in Chinese studies. Previous studies in Spain [15], Poland [8], and Austria [33] presented similar trends, suggesting that ethnic factors may influence maternal cholesterol levels. Regarding HDL‐C concentration, only a minor discrepancy was observed between our results and those reported in Chinese and European studies. HDL‐C is the lipid component exhibiting the least variability during the third trimester, as shown in Jimenez and Lockitch’s studies [15, 34]. Overall, our results are largely consistent with those reported in Chinese studies, supporting regional similarities in maternal lipid metabolism.

A complication associated with dyslipidemia in pregnancy is GDM [35]. GDM is a type of metabolic disorder of carbohydrate metabolism, in which there is an inadequate pancreatic β‐cell response to maternal insulin resistance. In the third trimester, elevated TG was consistently associated with GDM, evidenced by a large meta‐analysis of 292 studies [35]. Decreased insulin production in GDM patients contributes to higher TG level, as insulin activates lipoprotein lipase to promote TG uptake. Besides, lower LDL‐C and HDL‐C levels were found to be related to GDM in few studies [36–38]. One possible explanation of this phenomenon is the LDL‐C and TC‐lowering effect in the third trimester of a low‐fat diet in pregnancy, as discussed above [30]. Another potential mechanism is the contribution of increased cholesteryl ester transfer protein in cases of high TG levels. Consequently, the exchange of cholesterol ester within HDL and LDL with TG in TG‐rich lipoproteins is augmented [39], which exhibits decreased levels of LDL‐C and HDL‐C.

Our results indicate that the history of macrosomia is linked to increased maternal TG in the third trimester. This association, along with the correlation between LGA infants and higher maternal TG levels, suggests a potential mechanism underlying the increased prevalence of LGA infants in women with a prior history of macrosomia [40]. In addition, a history of HDP was a factor contributing to TG elevation. This finding may reflect long‐term changes in maternal metabolism after HDP, including increased insulin resistance, obesity, and adverse biochemical profile (higher TC, LDL‐C, TG, and glucose) [41, 42]. These residual metabolic changes could predispose women to higher TG level in subsequent gestations. In summary, TG levels appear to be an important link between maternal metabolic disorders and pregnancy outcomes.

However, participants in our study were healthy women without preexisting chronic diseases. As the first study on maternal lipid profile among Vietnamese pregnant women, we would like to establish the reference ranges and associated factors of lipid levels. Exclusion of preexisting chronic disease was to avoid the impact of pathological changes on the result, e.g., Type I diabetes was associated with decreased maternal TC and LDL‐C [43, 44]. Nevertheless, this approach may limit the generalizability of our findings to pregnant women with comorbidities. Future studies should include women with chronic diseases to better understand how comorbid diseases affect lipid metabolism during pregnancy.

This study has some strengths and limitations. We conducted the first study on the Vietnamese population regarding lipid levels in the third trimester. Our result can be used as a reference range for Vietnamese gravida. The large sample size, with relatively equal distribution across gestational age groups in the third trimester, is a significant strength of this study. To our knowledge, few studies in the third trimester displayed the number of participants in gestational age groups. We also presented the association between obstetric history and maternal lipid levels in the third trimester, which is rarely mentioned in previous studies. Besides, all blood sample measurements were performed within 2 h after sampling on one system. This method helped avoid the possible impact of measurement and the freeze–thaw process.

However, several limitations should be noted. First, a single measurement of serum lipid levels may not capture the intraindividual variability. Second, the maternal prepregnancy weight, a history of disease, and other basic data were self‐reported, and recall bias is difficult to avoid and can affect the results. Third, some lifestyle factors such as behavior, socioeconomic status, and physical activity, which are related to lipid levels, were not collected. Finally, this is a single‐center study with limited population diversity. A multicenter study with multiple populations across Vietnam would provide more representative results.

## 5. Conclusion

In conclusion, we estimated the 95% reference range of serum TC, LDL‐C, HDL‐C, and TG levels of healthy Vietnamese pregnant women in the third trimester. Complicated pregnancy presented with significantly lower levels of TC, LDL‐C, HDL‐C, and higher levels of TG than normal ones. We found common factors related to lipid levels such as prepregnancy BMI and gestational age. Additionally, obstetric complications including GDM, a history of HDP, and a history of LGA were associated with significant changes in maternal LDL‐C, HDL‐C, and TG concentrations.

## Funding

The authors have not received any financial support for this study.

## Conflicts of Interest

The authors declare no conflicts of interest.

## Data Availability

The data that support the findings of this study are available from the corresponding author upon reasonable request.
